# High-resolution synchrotron K-edge subtraction CT allows tracking and quantifying therapeutic cells and their scaffold in a rat model of focal cerebral injury and can serve as a reference for spectral photon counting CT

**DOI:** 10.7150/ntno.79575

**Published:** 2023-01-16

**Authors:** Clément Tavakoli, Elisa Cuccione, Chloé Dumot, Joëlle Balegamire, Salim Aymeric Si-Mohamed, Johoon Kim, Claire Crola-da-Silva, Yves Chevalier, Loïc Boussel, Philippe Douek, David Cormode, Hélène Elleaume, Emmanuel Brun, Marlène Wiart

**Affiliations:** 1 Univ Lyon, CarMeN Laboratory, INSERM, INRA, INSA Lyon, Université Claude Bernard Lyon 1, 69003, Lyon, France.; 2 Univ. Grenoble Alpes, Inserm UA7 Strobe, Grenoble, France.; 3 Hospices Civils de Lyon, Lyon, France.; 4 LAGEPP, University of Lyon 1, CNRS UMR 5007, 43 bd 11 Novembre, 69622 Villeurbanne, France.; 5 Department of cardiovascular and thoracic radiology, Louis Pradel Hospital, Hospices Civils de Lyon, 59 Boulevard Pinel, 69500 Bron, France.; 6 CREATIS, UMR 5220, Univ Lyon, INSA Lyon, University Claude Bernard Lyon 1, Lyon, France.; 7 Department of Radiology, University of Pennsylvania, Pennsylvania, United States.; 8 CNRS, Lyon, France.

**Keywords:** Synchrotron K-edge subtraction CT, spectral photon-counting CT, regenerative medicine, cell tracking, neurology

## Abstract

**Background:** The objective of this study was to demonstrate that synchrotron K-edge subtraction tomography (SKES-CT) can simultaneously track therapeutic cells and their encapsulating carrier, *in vivo,* in a rat model of focal brain injury using a dual-contrast agent approach. The second objective was to determine if SKES-CT could be used as a reference method for spectral photon counting tomography (SPCCT).

**Methods:** Phantoms containing different concentrations of gold and iodine nanoparticles (AuNPS/INPs) were imaged with SKES-CT and SPCCT to assess their performances. A pre-clinical study was performed in rats with focal cerebral injury which intracerebrally received AuNPs-labelled therapeutic cells encapsulated in a INPs-labelled scaffold. Animals were imaged *in vivo* with SKES-CT and back-to-back with SPCCT.

**Results:** SKES-CT revealed to be reliable for quantification of gold and iodine, whether alone or mixed. In the preclinical model, SKES-CT showed that AuNPs remained at the site of cell injection, while INPs expanded within and/or along the lesion border, suggesting dissociation of both components in the first days post-administration. Compared to SKES-CT, SPCCT was able to correctly locate gold, but not completely located iodine. When SKES-CT was used as reference, SPCCT gold quantification appeared very accurate both *in vitro* and *in vivo*. Iodine quantification by SPCCT was also quite accurate, albeit less so than for gold.

**Conclusion:** We here provide the proof-of-concept that SKES-CT is a novel method of choice for performing dual-contrast agent imaging in the context of brain regenerative therapy. SKES-CT may also serve as ground truth for emerging technologies such as multicolour clinical SPCCT.

## Introduction

Ischemic stroke affects 9.5 million persons each year in the world 1. The first line of treatment consists of re-establishing the brain circulation in the very first hours following onset. However, after this hyperacute window, there are currently no treatments available beside rehabilitation. Regenerative medicine is raising many hopes for treating ischemic stroke after the hyperacute stage. Cellular therapies are currently evaluated in Phase 0/1/2a clinical stroke trials 2. However, the optimal treatment regimen still needs to be determined, including cell type and dosage, therapeutic window, and the route of administration. Despite its invasiveness, the intracerebral route has been investigated in stroke patients, with promising results in terms of safety and potential efficacy 3-6. The main advantage of intracerebral injection of cells is a better control of the delivery to the target region; however, the downside is that the post-ischemic cerebral environment may not be a favorable host for cell survival. For enhancing *in situ* viability, therapeutic cells may be encapsulated within a bio-engineered scaffold before transplantation. The use of biomaterials to improve cell therapy after stroke must be carefully investigated in experimental studies prior to clinical transfer. In this respect, there is a crucial need to develop “bicolor imaging”, allowing the distinct and simultaneous *in vivo* monitoring of therapeutic cells on the one hand and cell-encapsulating scaffold on the other hand.

X-ray computed tomography (CT) is emerging as a promising tool for tracking cells labelled with metal-based contrast agents such as gold nanoparticles 7-9. However, with conventional CT systems, it is not possible to differentiate gold-labelled cells from other dense materials such as microcalcifications, or from bones if cells are found in their close vicinity. Synchrotron K-edge subtraction computed tomography (SKES-CT) is a robust method for the specific and quantitative imaging of contrast agents based on different elements, because monochromatic radiations can be used to directly measure the attenuation coefficients in CT images 10-12. Although this approach is not new, to the best of our knowledge, SKES-CT has not been used thus far for the specific detection and quantification of cells and/or scaffolds. In addition, methods that are used to track cells or scaffold in the whole brain of living rodents have an order of magnitude lower spatial resolution than SKES-CT (hundreds of µm vs tens of µm). In this context, the aim of this work was to provide the proof-of-concept that SKES-CT allows monitoring of therapeutic cells and their encapsulating scaffold simultaneously in a rat model of focal cerebral injury using a dual-contrast agent approach. Gold nanoparticles were used for labelling therapeutic cells and iodine nanoparticles were used for labelling scaffold. We evaluated the performance of SKES-CT for the quantification of gold, iodine and their mixture *in vitro* in phantoms, and *in vivo* in a preclinical study.

Because synchrotrons are not widely available, our next aim was to investigate whether SKES-CT may serve as a method of reference for translating this dual-contrast agent imaging approach to spectral photon-counting CT (SPCCT). SPCCT scanners use photon-counting energy resolved detectors, which improve the diagnostic value through a better signal-to-noise ratio. These next-generation of CT scanners are now available for human imaging, and clinical trials are underway to evaluate their added value over conventional and dual-energy CTs (8,9,13-15). Beside an improvement in image quality, one of the advantages of SPCCT is that it opens the possibility to perform specific and quantitative imaging of distinct contrast agents simultaneously (13-15). With the aim of clinical translation, we thus performed SPCCT back-to-back with SKES-CT 13. SPCCT allowed tracking of gold-labelled cells in the brain of rats having focal cerebral injury up to 2 weeks post-injection 13. In addition, SPCCT quantification of gold-labelled cells was in good agreement with that of ICP-OES both *in vitro* on phantoms and *in vivo* on collected organs 13. However, ICP-OES is a destructive technique that does not provide information about 3-dimensional distribution of contrast agents inside the brain. In the current paper, we thus compared SPCCT performance for the local detection and quantification of gold-labelled cells and iodine-labelled scaffold, using SKES-CT as a method of reference.

## Materials & Methods

### Contrast agents

#### Gold nanoparticles (AuNPs)

The AuNPs were synthesized for the purpose of the study according to a protocol described elsewhere 13, 14. They are composed of a 11 ± 1 nm gold core coated with 11-mercaptoundecanoic acid (11-MUDA). This coating favours cellular uptake of the nanoparticles because of its anionic properties and of the formation of a preferential protein corona 14. The resulting nanoparticles have a 22 nm hydrodynamic diameter and polydispersity index of 0.2 (*PdI* = [SD/mean]^2^). The zeta potential of 11-MUDA AuNPs is -44.4 mV at physiological pH and their peak absorbance is 524 nm. The 11-MUDA AuNPs were sterilized by syringe filtration (size: 0.45 µm) before further use.

#### Iodinated nanoparticles (INPs)

The INPs were specifically developed for imaging with SPCCT 15. They were synthesized according to a protocol described elsewhere 13, 15. They have a core size of 100 ± 15 nm, a 122 nm hydrodynamic diameter (*PdI* ≈ 0.2) and are densely coated with polyethylene glycol (PEG), which prevents them from fast elimination *in vivo* by macrophages. Their maximum UV absorbance is at 237 nm wavelength.

### Cells and scaffold labelling

Bone-marrow derived macrophages stimulated with interleukin-4 (for anti-inflammatory M2 polarization) were used as therapeutic cells 13. Cell labelling was obtained by overnight incubation of cells with AuNPs at 0.1 mg (Au)/mL. With this protocol, the gold loading per cell was 128 ± 34 pg (Au)/cell 13. Commercially available PuraMatrix (3-D Matrix, MA, USA) was used as the encapsulating scaffold. For scaffold labelling, INPs at 100 mg(I)/mL were simply mixed with PuraMatrix and therapeutic cells with a 1:8 ratio (12.5 mg(I)/mL).

### Phantom study

Three phantoms were designed to evaluate the quantification performance of the imaging methods. The first phantom, named “Bicolor phantom”, was designed to illustrate the principle of SKES-CT. It consisted of a set of twenty-four 0.5-mL centrifuge tubes containing either only 1% agarose (N=5); or contrast agents suspended in 1% agarose gel: 8.00 mg/mL of AuNPs (N=10), or 7.30 mg/mL of INPs (N=2); or a mixture of 4.00 mg/mL AuNPs and 3.65 mg/mL INPs (N=6). These concentrations were chosen so that attenuation images alone were not sufficient to identify the composition of the individual tubes. The tubes were positioned in the phantom to form specific patterns when imaged as gold and iodine-specific images.

Two other sets of phantoms were prepared with AuNPs and INPs with a range of gold and iodine concentration. The second phantom, named “AuNPs-labelled cell pellets”, consisted of a series of 5 tubes containing AuNPs-labelled cells in different quantities (0 - 1×10^6^ - 0.5×10^6^ - 0.25×10^6^ - 0.125×10^6^ cells) in 10-µL PBS. Cells were placed at the bottom of 1-mL Eppendorf tubes and fixed with 1% agarose gel on top. In the third phantom, named “INP-labelled gel”, INPs were suspended in 1% agarose gel in 1-mL Eppendorf tubes (N=11), with the following iodine concentrations: 0.00, 1.27, 1.90, 2.54, 3.81, 5.08, 7.61, 10.15, 12.69, 19.04, 25.38 mg/mL.

### Preclinical study

Data are reported according to the ARRIVE guidelines (Animal Research: Reporting of *In vivo* Experiments).

### Animals

Experiments involving animals and their care were carried out in accordance with the European regulation for animal use (EEC Council Directive 2010/63/UE, OJ L 276, Oct. 20, 2010). The study was approved by the local ethics committee of two institutions (Cermep: C2EA - 42, APAFIS agreement number #4688 and ESRF: ETHAX #113, APAFIS agreement number #7457). It involved 21 adult male Sprague-Dawley rats (Janvier, France; age at reception: 6-7 weeks, body weight: 250-300 g). The animals were housed in a temperature- and humidity-controlled environment (21 ± 3 °C), with a 12-hour light-dark cycle. The animals had free access to food and water and their cages were enriched with transparent red-coloured tunnels and hazelnut wood sticks. An acclimation period of at least 7 days was observed before the start of the study.

### Study design

**Figure 1:**
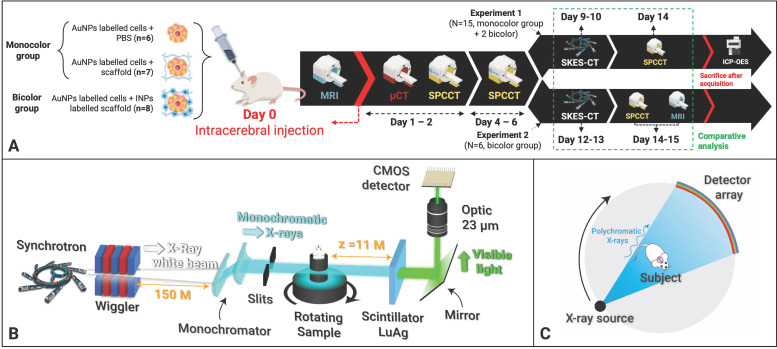
Experimental set-up and study design. **A:** Design of the preclinical studies. **B:** Synchrotron K-edge Imaging setup. **C:** SPCCT imaging setup.

Figure 1A shows the pre-clinical study design. In brief, a model of focal cerebral injury that mimics a chronic stroke cavity was induced in 21 rats by an intracerebral injection of 50 µg of lipopolysaccharide (LPS) from Escherichia coli (Sigma-Aldrich, Saint-Louis, USA) dissolved in 4 µL saline. Cell therapy was administered two weeks later (day 0) directly within the lesion using stereotaxic coordinates determined by a baseline magnetic resonance imaging (MRI). Rats were allocated to monocolor group (N=13, gold-labelled cells only) or bicolor group (N=8, gold-labelled cells + iodine-labelled scaffold). Treatment delivery was monitored by performing µCT (Siemens INVEON, Munich, Germany) immediately after intracerebral administration: rats were excluded in case of administration failure.

For the monocolor group, cell therapy consisted in a 10-µL volume of 0.5×10^6^ gold-labelled cells in PBS (N=6) or in unlabelled scaffold (N=7). Rats were imaged with SPCCT twice during the first week: at day 1 or 2 (D1/2) and at day 4, 5 or 6 (D4/5/6) post-transplantation. At day 7, rats were transported to the European Synchrotron (ESRF) and a 48-hours delay was respected for acclimation before imaging. Rat brains were then imaged *in vivo* with SKES-CT at day 9 or 10 (D9/10). Rats were imaged one last time on day 14 or 15 (D14/15) with SPCCT and MRI. At the end of the experiment, the rats were sacrificed, and their brains were prepared for ICP-OES analysis.

For the bicolor group, cell therapy consisted in a 10-µL volume of gold-labelled cells: 0.5×10^6^ (N=4), 0.25×10^6^ (N=2) or 0.125×10^6^ cells (N=2) in iodine-labelled scaffold. The imaging protocol was the same as for the monocolor group for 2 rats injected with 0.5×10^6^ cells. The remaining 6 animals were re-imaged *in vivo* by SKES-CT at day 12 or 13 (D12/13), followed by MRI and SPCCT on day 14 or 15 (D14/15).

### *In-vivo* imaging

For all imaging procedures, anaesthesia was initiated using 4% isoflurane. For MRI, SPCCT and µCT, rats were placed in prone position in a dedicated cradle and anaesthesia was maintained with 1-2% isoflurane during all the imaging procedure. For SKES-CT, rats were placed in an upright position on a home-made support and anaesthesia was maintained by an intraperitoneal injection of a ketamine (80 mg/kg bw)/xylazine (10 mg/kg bw) cocktail prepared in sterile water.

### MRI

MRI was performed using a 7T magnet (Avance II, Bruker Biospin, Ettlingen, Germany) equipped with 440 mT/m gradients and piloted with Paravision 5.0 software. A 72-mm diameter bird-cage coil was used for emission and a 25-mm diameter surface coil for detection. A T_2_-weighted spin-echo sequence was obtained with the following acquisition parameters: matrix 256×128, 35 slices with 800-µm slice thickness, TE/TR 57.7-ms/5000, RARE factor 8, duration 4 minutes.

### SKES-CT

Figure 1B shows the imaging set-up for SKES-CT and Table 1 summarizes system characteristics and acquisition parameters. Almost parallel, monochromatic X-ray beams (monochromatized by silicon crystals in Laue/Laue configuration) were tuned above or below the K-edges of interest. The detector placed 11 m downstream of the sample recorded the sinograms while the samples were rotated 360 degrees. The detector was a S-CMOS (PCO, Germany) coupled to an optic system (Optic Peter, France), resulting in a pixel size of 21.36 µm. Acquisitions above or below the K-edges of gold and iodine were recorded sequentially. Samples with gold only were scanned below (80.2 keV) and above (81.2 keV) gold K-edge (80.72 keV). Samples with gold and iodine were additionally scanned below (32.9 keV) and above (33.9 keV) iodine K-edge (33.17 keV).

### SPCCT

Figure 1C shows the imaging set-up for SPCCT and Table 1 summarizes system characteristics and acquisition parameters. The SPCCT system uses a polychromatic cone beam (W anode) and an energy selective detector with a resulting pixel size of 250 µm. Images were acquired in helical acquisition mode, with a tube current of 100 mA, a tube voltage of 120 kVp and energy bin thresholds of 30, 53, 78, 83 and 98 keV.

### Material Decomposition

Material decomposition process allows to obtain concentration maps of the elements of interest (gold and iodine), by mathematically combining the initial images. A specific data processing pipeline has been developed for each imaging method.

### SKES-CT

**Figure 2:**
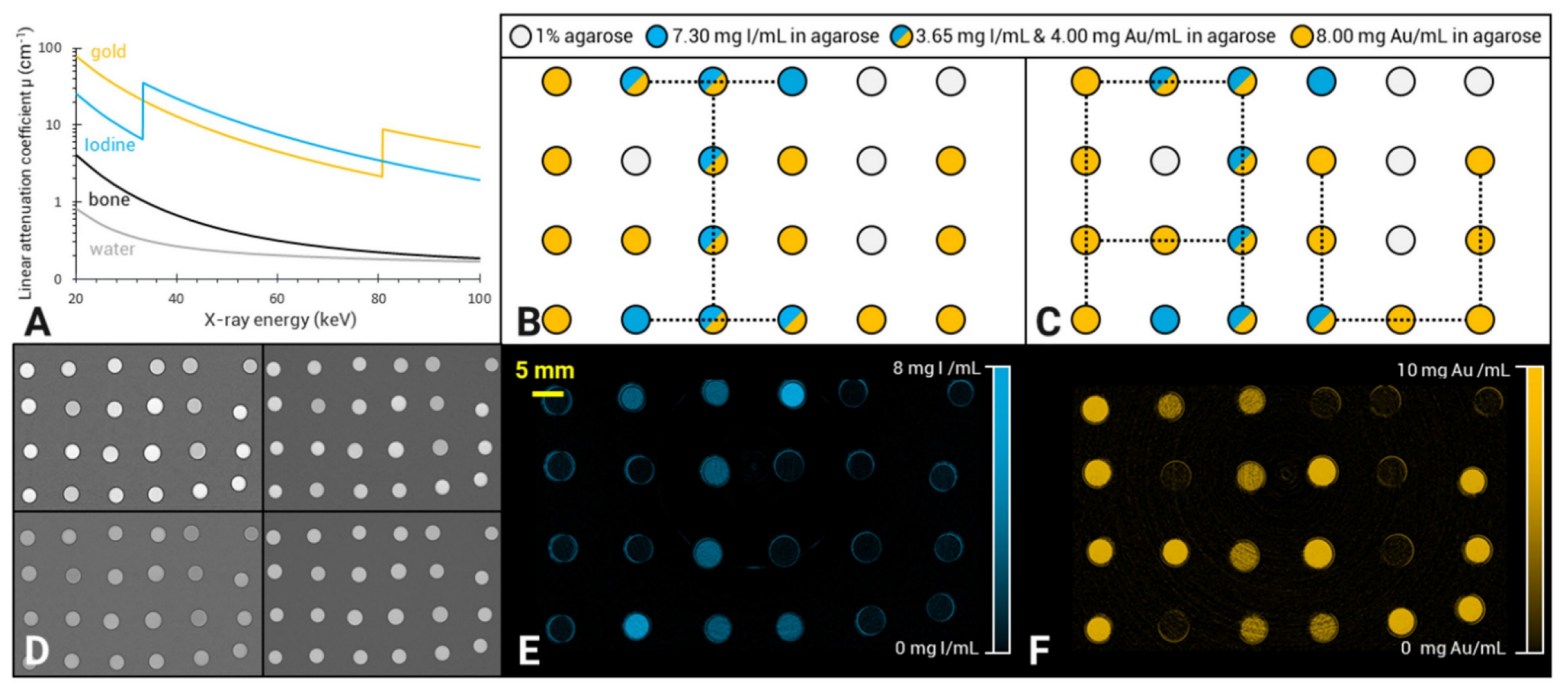
** SKES-CT allows specific quantification of gold, iodine and their mixtures *in vitro*. A:** Energy-dependent linear attenuation coefficients for gold, iodine, bone and water. Note the discontinuities at iodine (33.17 keV) and gold (80.72 keV) K-edges; **B-C:** Diagrams of the iodine (B) and gold (C) concentration in the tubes constituting the phantom: the light grey tubes contained 1% agarose; the blue only tubes contained 7.30 mg/mL of INPs in 1% agarose; the mixed blue and yellow tubes contained a mixture of 4.00 mg/mL AuNPs and 3.65 mg/mL INPs in 1% agarose and the yellow only tubes contained 8.00 mg/mL of AuNPs in 1% agarose. **D:** Conventional images acquired at 4 different energies, above and below both gold and iodine K-edges (top-left: 32.9 keV, top-right: 33.9 keV, bottom-left: 80.2 keV, bottom -right: 81.2 keV); **E:** SKES-CT Iodine concentration map. Note the I-shape letter that appears in blue; **F:** SKES-CT gold concentration map. Note the Au-shaped letters that appear in yellow.

SKES-CT uses monochromatic radiation (∆E/E = 0.1%) and takes advantage of the µ discontinuity around the K-edge of the contrast element of interest (Figure 2A). For material decomposition, the use of monochromatic radiation from a synchrotron simplifies the mathematical processing of the data, since the values read from the CT images are directly the linear attenuation coefficients of the mixture of elementary components in each voxel. Assuming that each voxel is composed of three elementary materials 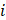 (G: gold, I: iodine and W: water), the linear attenuation coefficient of this mixture can be written as:



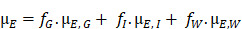



With 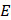: the energy of the X-ray beam [keV]


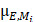
: the linear attenuation coefficient of the material 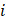 at the energy E [cm^-1^]


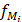
: the volume fraction occupied by the element 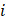 within the voxel

The concentration of the element 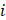 can be retrieved by multiplying the volume fraction of element 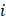 by its density:



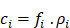



With 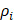 the element 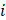 density [g.cm^-3^]

If only three elements compose the voxel of interest, the concentration of each element can be calculated from two CT images acquired at two different energies (E_1_, E_2_), by solving the system of 3 linear equations:


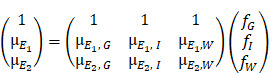

(Eq 1)

Such well-determined system can be solved by using matrix inversion algorithm which gives us 3 unique solutions for 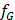, 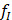 and 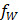. The accuracy of the measurement depends on several parameters including: the concentrations of the elements, the X-ray dose used for the image acquisition and the choice of the energies. When a high Z element is the element of interest, discontinuities in its linear attenuation coefficient can be used to improve its detection and quantification. As an example, the accuracy in the measurement of the concentration of gold will be maximised by acquiring two images above and below the gold K-edge. Note that the concentration of iodine can also be calculated for the data set acquired above/below the gold K-edge, but the accuracy of its measurement will be lower. When the sample contained iodine, we therefore repeated the acquisitions above/below the iodine K-edge for optimizing its quantification.

The use of a monochromator to adjust the synchrotron X-ray energy resulted in slightly different geometries, which induces a small shift between the images depending on the energy. Dedicated rigid registration algorithms were developed to superimpose the K-edge images before calculating the concentrations maps 16. In addition, we had to take into account the phase contrast that is observed in the images due to the large distance between the sample and the detector (11 m, Figure 1B), combined with the small pixel size used (21.36 µm) 17. In order to increase the Signal to Noise Ratio 18 on the linear attenuation coefficient, a phase retrieval method 19 was applied prior to tomographic reconstruction.

### SPCCT

In contrast to synchrotron, SPCCT uses conventional polychromatic X-ray source and dedicated energy-resolving detectors, known as photon-counting detectors (PCD), which allow the measurement of each incoming photon energy, based on the energy bin they belong to (6 energy bins were used in this study) (Figure 1C). To optimize the accuracy of the material decomposition by SPCCT it is advantageous to set the energy bin limits near the K-edges of the elements of interest 20. This method was implemented in this study by setting the energy bins thresholds at 30, 53, 78, 83 and 98 keV. With SPCCT, quantification of the elementary materials is made possible by using a two-step material decomposition approach, which consists of first generating material-specific sinograms using a maximum likelihood algorithm and then reconstructing them into material-specific images 21. In this study, images were decomposed in gold, iodine and water maps.

### Image analysis

In the phantom studies, the content of the tubes was segmented using simple visual thresholding on the attenuation images for both imaging modalities. Gold and iodine concentrations were measured in resulting ROIs on corresponding concentration maps.

For the monocolor groups, SPCCT images acquired at D4/5 post-transplantation were compared to SKES-CT images acquired 5 days later, at D9/10 (Figure 1A). For the bicolor group, SKES-CT images acquired at D12/13 were compared to SPCCT images acquired 2 days later, at D14/15, except for the two rats that had the same acquisition protocol as for the monocolor study and that were analyzed the same way, *i.e.,* SPCCT at D4/5 vs SKES-CT at D9/10.

Quantitative analysis of the *in-vivo* concentration maps was performed after registration of SPCCT onto the SKES-CT volumes. Briefly, a rigid registration of the cranial skull was performed with a gradient descent method using a mean squared difference metric. Regions containing gold or iodine were automatically segmented in SKES-CT images restricted to the brain region using a concentration threshold set at 2 mg/mL. This threshold was used because it was considered as the SPCCT detection limit 22. In addition, to limit the impact of noise, we filtered out the structures smaller than eight voxels. These segmentation masks were then binned to match SPCCT spatial resolution and used for quantification on gold or iodine-specific maps. We calculated the gold or iodine mass per voxel by multiplying the element concentrations by the voxel volume and finally obtained the total mass by adding the masses of each voxel in the segmented region.

### Statistics

Data are presented as mean ± standard deviation within samples or within regions of interest when one sample is considered. A linear regression model was used to correlate the gold and iodine mass estimated by two different methods. The agreement between SPCCT and SKES-CT for gold and iodine quantification *in vivo* was assessed with Bland-Altman plots.

## Results

### Phantom study

The iodine (Figure 2B) and gold (Figure 2C) contrast agents of the “Bicolor phantom” cannot be distinguished from each other based on conventional images, as shown on Figure 2D. These four images were acquired below and above iodine and gold K-edges for SKES-CT. Material decomposition provides element-specific concentration maps labelled with an “I letter” (Figure 2E) and “Au letters” (Figure 2F). Visual identification of gold and iodine was obtained even when they were mixed in the same tube (vertical line in the I letter for instance corresponding to the last vertical line of the A letter). SKES-CT quantification of gold and iodine concentrations was highly consistent in both monocolor and bicolor tubes (Table 2).

**Figure 3:**
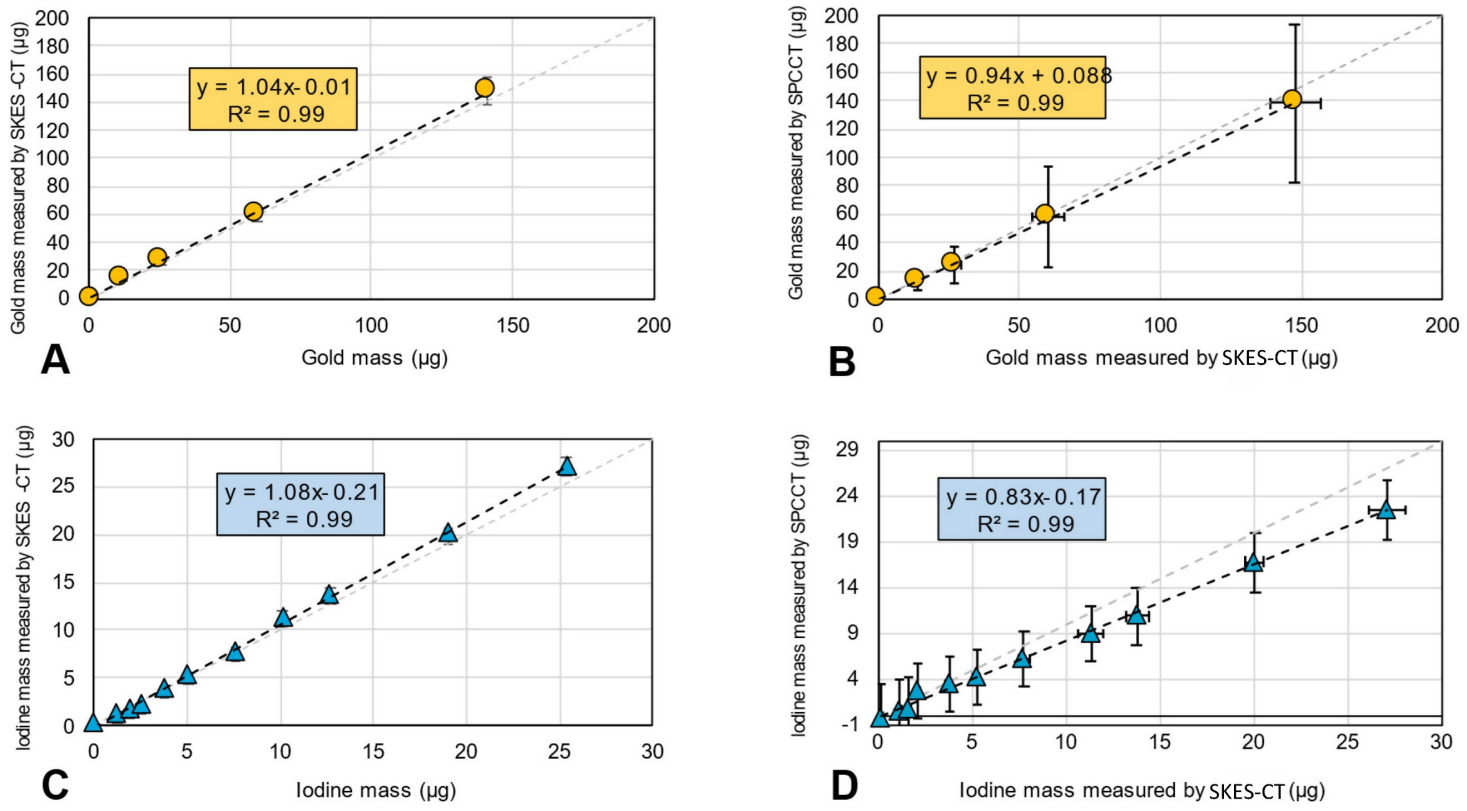
** SKES-CT accurately quantifies gold and iodine and may serve as a method of reference for SPCCT *in vitro*. A-B:** Gold mass estimation in “AuNP-labelled cell pellets” using SKES-CT (A) and SPCCT (B). **C-D:** Iodine mass estimation in “INP-labelled gel” using SKES-CT (C) and SPCCT (D).

In the “AuNPs-labelled cells” and “INP-labelled gel” phantoms, gold and iodine were also accurately quantified, with a slope of 1.04 and 1.08, respectively, and R^2^ of 0.99 for both elements (Figure 3A for gold and Figure 3C for iodine). There was also a linear relationship between the quantification of gold mass in cell pellets obtained by SPCCT and SKES-CT (R² = 0.99, Figure 3B), although SPCCT slightly underestimated gold mass by 6% relative to the synchrotron reference measurement (slope of 0.94, intercept of 0.1 µg). For iodine, quantification by SPCCT was underestimated by 17% compared to SKES-CT (slope of 0.83, intercept of - 0.1 µg), albeit with a linear relationship (R² = 0.99, Figure 3D). These two phantoms were scanned prior to each SPCCT acquisition session and the SPCCT *vs* gold or iodine concentration curves were used as a calibration to correct gold and iodine measurements obtained with this modality in the pre-clinical study.

### Preclinical study

Three animals were excluded from the study: one because it died on the day of injection, one because of administration failure, and one because of technical problems with SPCCT, resulting in 10 animals in the monocolor group. One rat from the bicolor group died at day 12 and was imaged post-mortem with both modalities.

**Figure 4:**
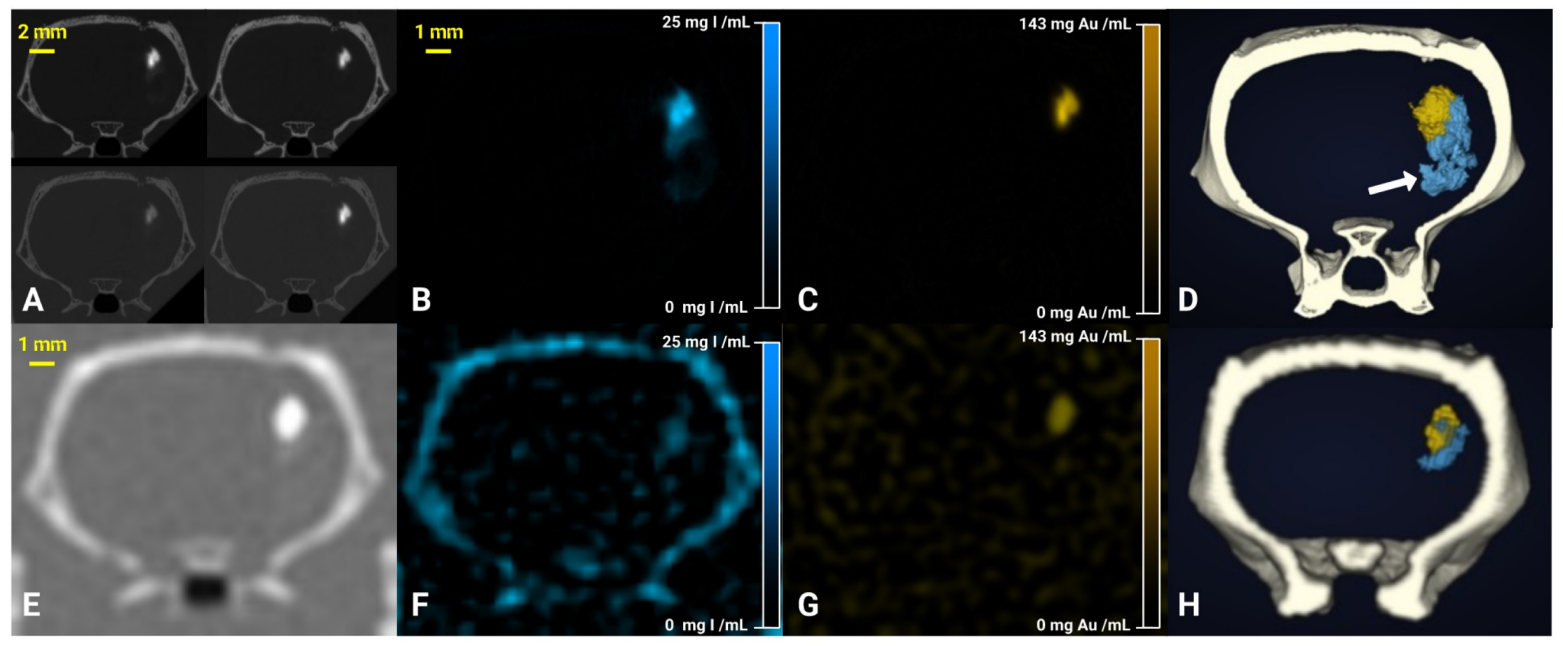
SKES-CT allows specific imaging of gold, iodine and their mixtures *in vivo* and may serve as a method of reference for SPCCT: back-to-back imaging of rat brain with a focal cerebral injury post-transplantation of AuNP-labelled macrophages embedded in INP-labelled scaffold using SKES-CT (A-D) and SPCCT (E-H). **A:** Synchrotron conventional images acquired at 4 different energies, above and below gold and iodine K-edges (top-left: 32.9 keV, top-right: 33.9 keV, bottom-left: 80.2 keV, bottom-right: 81.2 keV). **B:** SKES-CT iodine concentration map. **C:** SKES-CT gold concentration map. **D:** Synchrotron SKES-CT 3D view of segmented bone (white), iodine (blue) and gold (yellow). **E:** SPCCT conventional image. **F:** SPCCT iodine concentration map. **G:** SPCCT gold concentration map. **H:** SPCCT 3D view of segmented bone (white), iodine (blue) and gold (yellow). White arrow on D shows iodine detected by SKES-CT and not by SPCCT.

Figure 4A shows conventional SKES-CT images acquired above and below the K-edges of gold and iodine in a typical rat. As for the “Bicolor phantom”, the iodine and gold contrast agents cannot be distinguished from each other based on these images alone. In contrast, iodine and gold concentration maps (respectively Figure 4B and 4C) revealed the distinct distribution of each contrast agent. In addition, the 3D representation of both contrast agents after maps segmentation allows to visualize how they are superimposed in some regions and separated in others (Figure 4D). One rat did not have gold signal thus suggesting that cell administration had failed (Figure 6A). In all other rats, the gold signal remained at the site of cell injection, while the iodine signal tended to expand within and/or along the lesion border as seen when element-specific maps were overlaid on MRI (Figure 6A-D), thus suggesting a dissociation of the two components of regenerative therapy. Of note, main anatomical landmarks such as the lateral ventricles and the oedematous cavity could be readily identified on SKES-CT images (Supplemental Figure 1). The average volume of segmented ROIs for gold quantification was 3 ± 2 µL (N=18, pooled monocolor + bicolor study). The average gold mass measured by SKES-CT was 41 ± 24 µg in rats injected with 500,000 gold-labelled cells (monocolor study, N=10), as compared to 48 ± 18 µg estimated by ICP-OES in the same rats. For iodine quantification, the average segmented volume in SKES-CT maps was 6 ± 3 µL (N=8, bicolor study only). In average, the iodine content estimated by SKES-CT was 38 ± 23 µg, as compared to 125 µg injected.

Figure 4E shows conventional SPCCT images obtained in rat shown in Figure 4A-D. Figure 4F-G shows element-specific maps (iodine and gold) and Figure 4H shows the segmentation of iodine and gold signals in 3D. As for SKES-CT, gold can be distinguished from iodine, in the iodine and gold concentration maps only. SPCCT concentration maps were noisier than those obtained with SKES-CT (for instance, sigma in brain parenchyma = 2.81 mg/mL for SPCCT and 0.39 mg/mL for SKES-CT in gold concentration images, respectively, Figure 4C and 4G). On visual assessment, SPCCT properly localized gold (Figure 4G vs Figure 4C), while it missed part of iodine compared to SKES-CT (Figure 4F vs Figure 4B and Figure 4H vs Figure 4D arrow).

SPCCT quantification of gold mass was in good agreement compared to SKES-CT (R² = 0.94, slope of 0.95 and bias of 3 µg with limits of agreement [-12; +15] µg, Figure 6A-B), over the range studied (0-140 µg). Furthermore, the presence of iodine in a voxel containing gold did not impact SPCCT gold quantification: belonging to the bicolor or monocolor groups had no effect on performances, as seen in Figure 6A-B. For iodine mass measurement, SPCCT measurements were more dispersed than gold data compared to SKES-CT (R² = 0.59, slope of 1.08 and bias of -9 µg with limits of agreement [-40; +26] µg) (Figure 6C-D).

## Discussion

**Figure 5:**
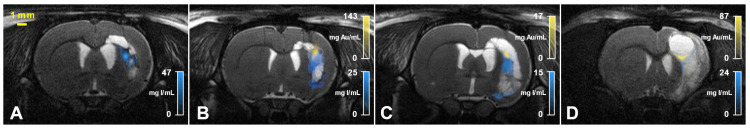
SKES-CT may be coupled with MRI to visualize the regenerative therapy components in relation to the cerebral lesion location: overlay between T2-weighted MRI and both iodine (blue) and gold (yellow) concentration maps obtained with SKES-CT for 4 representative rats of the bicolor study (**A-D**).

**Figure 6:**
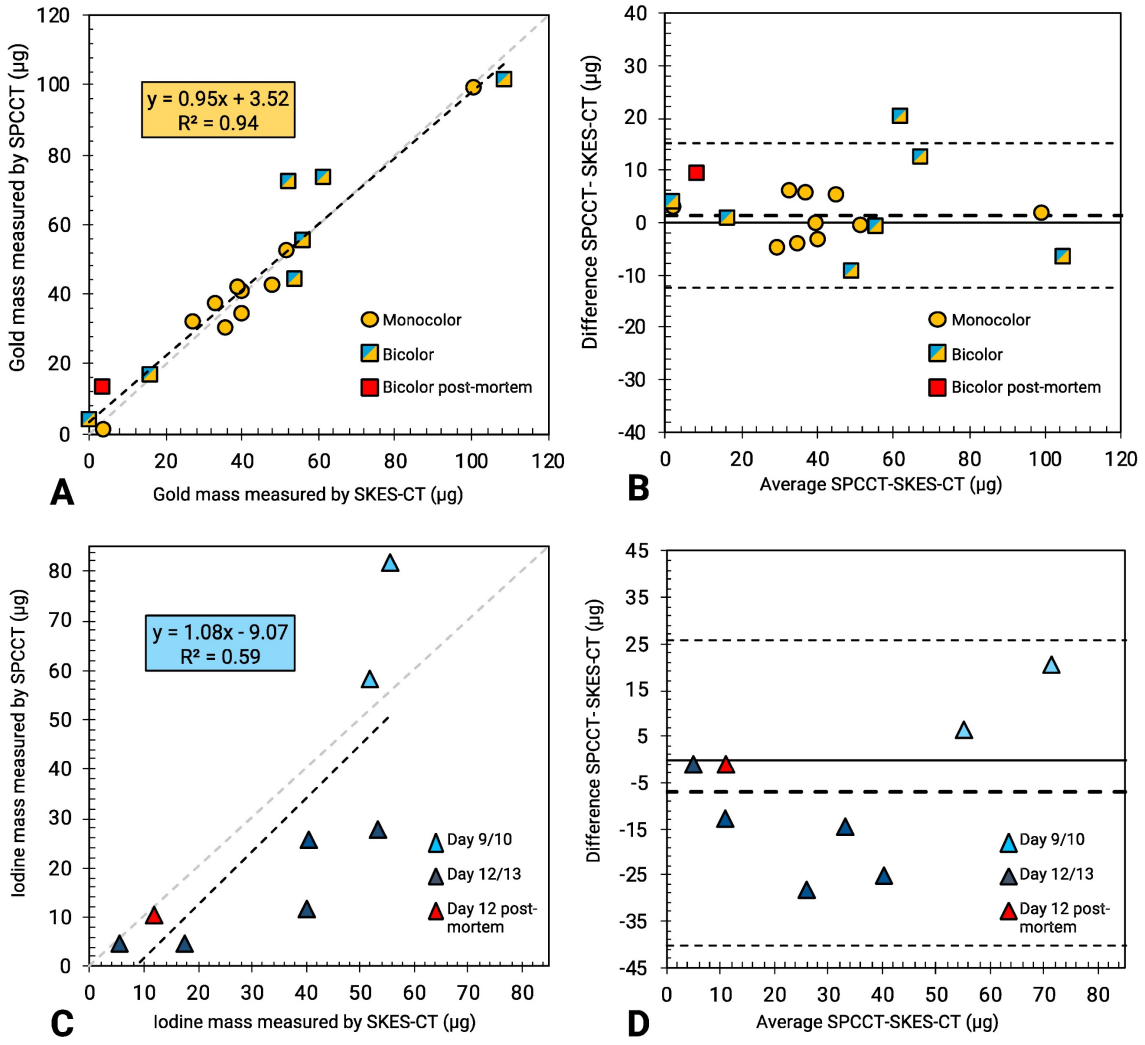
** SKES-CT may serve as a method of reference for SPCCT *in vivo*. A:** Linear relationship between SKES-CT and SPCCT gold quantification for both monocolor and bicolor studies (red square: one rat died and was imaged post mortem using both modalities). **B:** Bland-Altman plot of SKES-CT and SPCCT K-edge gold quantification for both studies. **C:** Linear relationship between SKES-CT and SPCCT material decomposition iodine quantification for the bicolor study. **D:** Bland-Altman plot of SKES-CT and SPCCT material decomposition iodine quantification for bicolor study. The days indicated are those of SPCCT imaging.

In this study, we demonstrate for the first time that SKES-CT may be used as a theranostic tool in a rat model of focal cerebral injury in the context of regenerative medicine. To the best of our knowledge, this study is the first one to report the use of bicolor SKES-CT for monitoring a combination of therapeutic cells and scaffold labelled with two distinct contrast agents. This enabled to observe the partial disassociation of cells and scaffold during the second week after their common *in situ* administration (Figure 5). The extent of cell and scaffold disassociation was unexpected, although some authors have reported that cells and scaffold mixture was not always homogenous throughout the lesion 23. The extent of disassociation was variable from one animal to another. This is important to assess such variability because these differences in *in vivo* behaviours might explain why some subjects respond to therapy while others do not. Future works should investigate whether labelling with gold and iodine had an impact on this disassociation using control unlabelled groups. The main advantages of SKES-CT are that 3-dimensional images of the whole brain are obtained with an acquisition time (12 minutes) that is compatible with rodent anaesthesia duration, at a very high spatial resolution (21 µm isotropic) and with an exquisite contrast. This allows for accurate localization of the contrast agents and facilitates co-registration with other 3-dimensional complementary imaging modalities such as MRI (Figure 5). In addition, main anatomical landmarks are readily seen, so that it is easy to orientate in the brain without the need for a complementary imaging modality (Supplementary Figure 1). The other main advantage of SKES-CT over other methods (MRI, optical imaging) is its excellent quantitative capabilities, as shown in the current paper with phantoms. In the pre-clinical study, SKES-CT provided values of iodine content that were much lower than what had been injected. This may be due to the fact that iodine nanoparticles were simply mixed with the scaffold for labelling and might thus have been rapidly eliminated from the brain, as previously reported in cardiac studies 24. To overcome this problem, we are currently working on stabilizing scaffold labelling with the help of innovative chemistry approaches. Both *in vitro* and *in vivo* results also indicate that quantification is not affected by the presence of two distinct contrast agents in the same voxel, thus confirming the value of SKES-CT for dual-contrast agent approaches.

Another asset of SKES-CT is the fact that it may be used in patients 25, making it a translational imaging tool. However, there are a number of obstacles to the application of SKES-CT in the clinical field. Firstly, patients have to be installed in a rotating chair to obtain tomographic data, as the synchrotron beam is a fan-shaped fixed beam. Secondly, synchrotrons are large in size and not readily available, which limits their widespread use in clinical trials. Therefore, another relevant application of SKES-CT would be to foster the development and validation of alternative, clinically-applicable imaging techniques, by serving as an *in vivo* ground truth.

Recently, a definite progress in X-ray computed tomography (CT) technology occurred with the introduction of spectral photon-counting CT (SPCCT) 26. This new technology paves the way to translate dual-contrast agent approaches into the X-ray clinical field 27-30. We here provide the first *in vivo* comparison of SPCCT with SKES-CT taken as a method of reference. This is actually very challenging because these two imaging modalities rely on totally different acquisition strategies and post-processing algorithms, thus resulting in reconstructed images with different spatial resolution, slice thickness, contrast-to-noise ratio and limits of detection. In particular, the SPCCT prototype that we have used was conceived as a clinical scanner except for its reduced field of view (15 cm), hence it has a rather low spatial resolution for rodent brain imaging (250 × 250 × 250 µm voxel) 31.

In phantoms, SPCCT performed as well as SKES-CT for gold quantification. For iodine, the current phantom study showed that there was an ~20% underestimation with SPCCT compared to SKES-CT. This is consistent with phantom studies performed with the same SPCCT prototype that used ICP-OES as a method of reference 15, 32. The reason for this underestimation may be related to the low energy level of iodine K-edge and the few numbers of photons crossing the sample below this energy. To account for this system shift, *in vivo* SPCCT data were corrected with *in vitro* SPCCT calibration curves.

In the pre-clinical study, there was a good visual co-localization between SPCCT and SKES-CT signals of gold and iodine, although SPCCT failed to detect some of the iodine, in particular when the scaffold expanded away from the injection site. This scaffold expansion resulted in a decrease of iodine concentration per voxel and a loss of signal on the SPCCT iodine-specific maps probably because of partial volume effects. For quantification, SPCCT revealed to be very accurate in estimating gold mass locally when compared to SKES-CT (32, 33). Interestingly, in the rat that was previously considered as an outlier because it had an abnormally high gold concentration in the brain (square with the highest concentration in Figure 5A) 13, SKES-CT here confirmed that the gold value estimated by SPCCT was in the right order of magnitude, thus showing the value of using SKES-CT rather than ICP-OES as a method of reference.

Iodine mass was less reliably assessed than gold mass by SPCCT, even after correcting for the system shift. There is therefore a need for improvement in scaffold detection and quantification with SPCCT. One of the possibilities would be to use a contrast agent with a higher Z-number than iodine, for instance gadolinium or ytterbium 21. For *in vivo* quantification, a detection threshold of 2 mg/mL was used to automatically generate a region of interest on SKES-CT that was then applied to SPCCT for both contrast agents. One of the perspectives of this work would be to evaluate the performances of SPCCT when it stands on its own, *i.e.* by performing the detection task on SPCCT metal-specific maps prior to quantification.

In summary, we have provided the proof-of-concept that SKES-CT was a novel method of choice for performing dual-contrast agent imaging in the context of cell therapy. SKES-CT may also serve as a ground truth for emerging technologies such as multicolor clinical SPCCT, in both pre-clinical and clinical studies. This opens the way to potential clinical translation of innovative X ray-based applications for cellular and molecular imaging. This study was performed in a rat model of focal brain injury but cell therapies extend well beyond neurological applications. SKES-CT may thus help designing and validating cell therapies in a range of chronic diseases, from ischemic stroke to osteoarthritis for instance.

## Supplementary Material

Supplementary figures.

## Figures and Tables

**Table 1:**
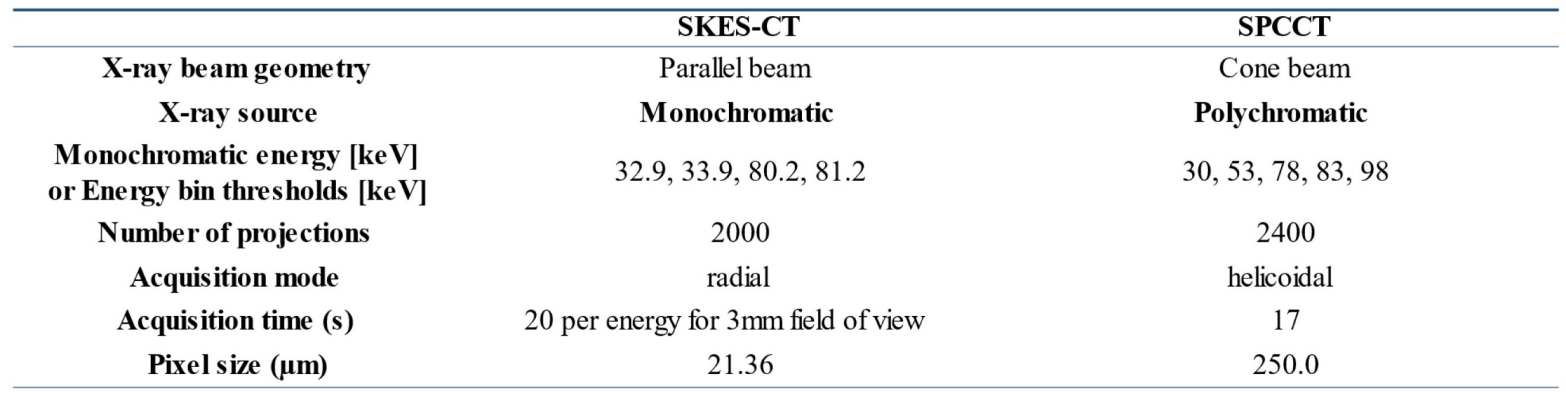
System characteristics and acquisition parameters for SPCCT and SKES-CT

**Table 2:**
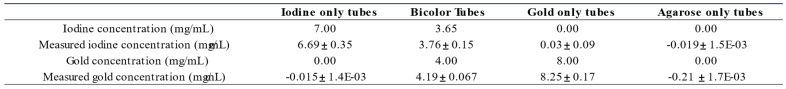
SKES-CT quantification of gold and iodine in the “Bicolor phantom”
