# Supplementary material for: High-resolution synchrotron K-edge subtraction CT allows tracking and quantifying therapeutic cells and their scaffold in a rat model of focal cerebral injury and can serve as a reference for spectral photon counting CT
Source: Nanotheranostics. 2023 Jan 16;7(2):176–86. doi: 10.7150/ntno.79575 (PMC9925349; doi:10.7150/ntno.79575)
Supplement: Supplementary file 1 — Supplementary figures. [file ntnov07p0176s1.pdf]

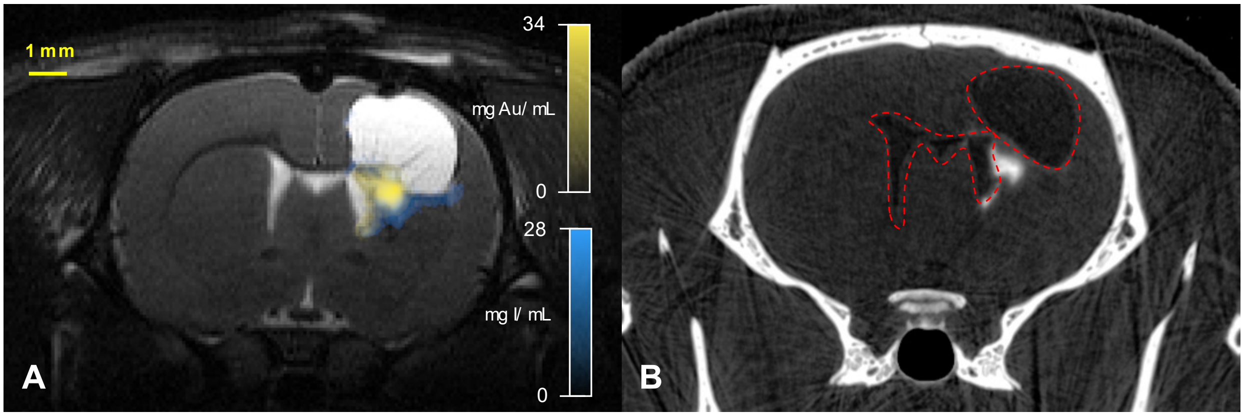

Figure S1: Synchrotron phase contrast images detect main anatomical landmarks including oedema. A: Overlay between T2-weighted MRI and both iodine (blue) and gold (yellow) concentration maps obtained with SKES-CT for one rat of bicolor study. B: Synchrotron attenuation image of the same rat with lateral ventricles and the oedematous cavity circled in red.
